# Metabolites Evaluation, Cytotoxicity, and Teratogenicity Analysis of Selected Medicinal Plants

**DOI:** 10.1155/sci5/5233425

**Published:** 2025-12-18

**Authors:** Mary Jhane G. Valentino, Tutik Sri Wahyuni

**Affiliations:** ^1^ Airlangga Post Doctoral Fellow, Universitas Airlangga, Surabaya, Indonesia, unair.ac.id; ^2^ Department of Biological Sciences, College of Science, Central Luzon State University, Science City of Munoz, Nueva Ecija, Philippines; ^3^ Department Pharmaceutical Science, Faculty of Pharmacy, Airlangga University, Surabaya, Indonesia, unair.ac.id; ^4^ Natural Product Medicine Research and Development, Institute of Tropical Disease, Airlangga University, Surabaya, Indonesia, unair.ac.id

**Keywords:** cytotoxicity, medicinal plants, phytochemicals, teratogenicity

## Abstract

The study determined the cytotoxicity and teratogenicity of 28 species of medicinal plants. The phytochemical properties and antioxidant activity were also evaluated. Plant materials were extracted using ethanol as solvent. Brine shrimp assay was performed for cytotoxicity test and zebra fish embryo‐based teratogenicity testing. The phytochemical constituents were detected using thin‐layer chromatography, while the total phenolics and antioxidant activity were determined using the Folin–Ciocalteu method and DPPH free radical scavenging assay, respectively. Results of phytochemical screening revealed the presence of 12 phytochemicals in the selected medicinal plants, which include essential oils, triterpenes, phenols, fatty acids, sugar, anthraquinones, coumarins, anthrone, tannins, flavonoids, steroids, and alkaloids. Total phenolic content which ranges from 125.45 to 568.99 GAE/g and antioxidant activity of 40.25%–82.56% were recorded from *M. calabura* and *Z. americana*, respectively. For the cytotoxicity test, *M. paniculata* registered the least LC_50_ of 1157.42 ppm, while *C. asiatica* has the highest LC_50_ at 33,252.69 ppm. Meanwhile, for the teratogenicity testing, various morphological abnormalities and teratogenic effects were observed in different developmental stages of zebrafish, which are lethal and sublethal, such as coagulation, yolk sac, and pericardial edema, and malformations (head, spines, and tail), growth retardation, restricted movement, and pigmentation. The LC_50_ values for teratogenicity suggest low to nonteratogenicity of the plant extracts with values ranging from 324 ppm in *A. brasiliana* to 11,933 ppm in *P. cablin*.

## 1. Introduction

Many plant species remain poorly studied and may harbor unique secondary metabolites [1]. Indigenous and local communities have long used native plants for medicinal purposes. Scientific studies are necessary to validate traditional practices and reveal bioactive compounds with therapeutic potential [2]. The chemical diversity found in this underexplored flora may lead to the discovery of compounds with unique modes of action that can offer new treatments, particularly valuable in addressing drug resistance and emerging diseases [3]. Philippines and Indonesia are recognized as biodiversity hotspots with high levels of endemic plants. In the Philippines, there are about 1500 medicinal plants, while in Indonesia about 2000–7500 species are used for medicinal purposes [4, 5].

The utilization of certain medicinal plants has facilitated the identification and isolation of therapeutic agents employed in addressing various human ailments [6]. Phenolic compounds, inclusive of tannins and flavonoids, are recognized for their antioxidant properties in both medicinal and culinary plants [7, 8]. Anthraquinones and anthrones have diverse biological activities, including anticancer and antimicrobial properties [9]. Moreover, coumarins suggest therapeutic applications in managing inflammatory conditions, cardiovascular diseases, and oxidative stress‐related ailments [10]. Steroids underscore the plants’ potential in treating inflammatory conditions, autoimmune disorders, and cancer [11]. This implies comprehensive phytochemical investigations for elucidating the therapeutic potential of various plants.

The brine shrimp cytotoxicity assay is a rapid bioassay that estimates general cytotoxicity of crude plant extracts by measuring *Artemia salina* nauplii mortality [12]. Based on studies, there is a correlation between brine shrimp and cytotoxicity in mammalian cell lines for many compound classes, while noting that correlation strength varies with extract complexity. On the other hand, zebrafish (*Danio rerio*) embryo teratogenicity assay provides a vertebrate model for teratogenicity assessment. The rapidly developing embryos allow direct observation of abnormalities, malformations, and developmental delays. These assays form an efficient and ethical procedure for the evaluation of potentially harmful bioactive compounds of the selected medicinal plants [13, 14].

In this study, 28 species of plants were selected based on their traditional use among the communities of Indonesia and Philippines, such as plant species from family *Cucurbitaceae* (*Coccinia grandis* (L.) Voigt, *Melothria pendula* Cogn.), *Passifloraceae* (*Passiflora foetida* L.) *Zingiberaceae* (*Kaempferia angustifolia* (Wall.) K. Schum.)*, Zingiber amaricans* Blume, *Boesenbergia pandurata* (Lour.) M.F. Newman and Skornick, *Curcuma heyneana* Valenton*, Curcuma zedoaria, Curcuma mangga* Valeton, *Zingiber zerumbet (*L.) Smith*, Curcuma zanthorriza* Roxb.), *Caprifoliaceae* (*Valeriana officinalis* L.), *Malvaceae* (*Sida rhombifolia* L.), *Lamiaceae* (*Pogostemon cablin* (Blanco)Benth. ex Merr.), *Piperaceae* (*Piper crocatum* Ruiz and Pav.), *Muntingiaceae* (*Muntingia calabura* L.), *Petiveriaceae* (*Rivina humilis* L.) and *Apiaceae* (*Centella asiatica* (L.) Urban), *Moraceae* (*Morus rubus* L.), *Rutaceae* (*Murraya paniculata* (L.) Jack), *Amaranthaceae* (*Alternanthera brasiliana* (L.) Kuntze), *Asteraceae* (*Sonchus arvensis* L.), *Talinaceae* (*Talinum paniculata* (Jacq.) Gaertn.), *Fabaceae* (*Acacia mangium* (Wild.) Wild.), *Verbenaceae* (*Lantana camara* L.), *Oleaceae* (*Nyctanthes arbor-tristis* L.), *Acanthaceae* (*Ruellia simplex* C. Wright, *Ruellia tuberosa* L*.), Rutaceae* (*Murraya paniculata* (L.) Jack), and *Oleaceae (Nyctanthes arbor-tristis* L.).

The evaluation of metabolites, cytotoxicity, and teratogenicity in selected medicinal plants provides information on the presence of various phytochemicals with known therapeutic potential. The cytotoxicity test using brine shrimp assay and teratogenic testing will screen the potential toxicological effects of the selected medicinal plants.

## 2. Experimental

### 2.1. Collection of Plants

Plant samples were collected from the Central Luzon State University, Nueva Ecija and Surabaya, Indonesia. Only healthy plants (leaves, roots, and rhizomes) devoid of any visible signs of illness, such as spotting, wilting, powdery mildew, or necrotic lesions, were selected. Plant samples were shade‐dried and ground into powdered form.

### 2.2. Preparation of Ethanolic Extraction

Fifty grams of the powdered plant parts (leaves, roots, and rhizomes) were soaked in 200 mL of 80% ethanol at ambient temperature. Extraction was carried out using a rotary evaporator [15].

### 2.3. Phytochemical Analysis

Phytochemical screening was conducted on each plant extract to identify the secondary metabolites present. The phytochemical screening followed a specific protocol. Each plant extract was applied as spots on marked and labeled plates, then subjected to thin‐layer chromatography (TLC). The development of the 7 × 4‐cm plates occurred in a solution of acetate‐methanol (7:3) at a depth of 16 mm. The spots representing certain metabolites were observed on the TLC plates, exposed to UV light and a hot plate to ensure proper separation of various compounds.

Vanillin‐sulfuric acid reagent was used in determining the presence of phenols, sterols, triterpenes, and essential oils. Methanolic potassium hydroxide was utilized for testing anthraquinones and anthrones, while phenolic compounds and tannins were detected by using potassium ferricyanide–ferric chloride reagent. Dragendorff’s reagent was employed to detect alkaloids, and Antimony III chloride for flavonoids [16].

### 2.4. Determination of Total Phenolic Content

The determination of total phenolic content in the ethanol extract was done using the Folin–Ciocalteu method. Specifically, 0.5 mL of ethanol extract (at a concentration of 10 μg/mL) was combined with 2.5 mL of Folin–Ciocalteu reagent. Following a reaction period of 2 min, 2.5 mL of 7% sodium carbonate solution was added to the mixture. The reaction mixture was then incubated at 30°C for 30 min to ensure completion. Absorbance was subsequently measured at 760 nm, using a blank as the reference. Gallic acid served as the standard for this procedure. The total phenolic content in the ethanol extracts was quantified and expressed in terms of gram gallic acid equivalents (gGAE) per 100 g of extract.

### 2.5. 1, 1‐Diphenyl‐2‐Picryl Hydrazyl (DPPH) Free Radical Scavenging Assay for Antioxidant Detection

The antioxidant activity was assessed using the DPPH radical scavenging assay, as outlined by Baliyan et al. [17]. This method involved evaluating the extract’s free radical scavenging activity (RSA) employing the DPPH technique. A stock solution was prepared by dissolving 24 mg of DPPH in 100 mL of methanol. For the assay, a standard solution comprising 3 mL of DPPH and 100 μL of methanol was placed in a test tube and incubated in complete darkness for 30 min. Absorbance was measured at 517 nm. The percentage of antioxidant activity or RSA was calculated using the following formula:

(1) % of antioxidant activity=Ac−As÷Ac×100.

Here, Ac represents the absorbance of the control (DPPH solution without extract), and As denotes the absorbance of the test sample (mixture of DPPH, ethanol, and crude extract). Catechin, a synthetic antioxidant, was used as a positive control.

### 2.6. Brine Shrimp (*A. salina*) Cytotoxicity Assay

Cytotoxicity refers to the harmful effects induced by the action of chemotherapeutic agents on living cells. In the realm of nanoparticles, cytotoxicity tests hold significant importance as they aid in assessing the suitability of proposed biomedical applications [18]. The brine shrimp eggs were sourced from the Biotechnology and Analytical Laboratory. These eggs underwent a rehydration process with distilled water lasting for 30 min. Subsequently, they were moved to a 1‐L plastic bottle filled with filtered sterile seawater. The seawater was prepared by dissolving 30 g of sea salt per 100 mL of water. The hatching of the shrimp occurred under well‐lit and aerated conditions for a duration of 24 h. The hatchery is covered with thin gauze for protection against undesirable animals and insects.

Plant ethanolic extracts was emulsified by incorporating dimethyl sulfoxide (DMSO). Serial dilutions (5000, 1000, 500, and 100 ppm) were then performed. Ten nauplii were gathered and transferred to the test tubes using a glass dropper. Subsequently, sterile seawater was added to each test tube to achieve a total volume of 3 mL. The number of surviving nauplii was observed under dissecting microscope and tallied at intervals of 12, 24, 36, and 48 h. The LC_50_ was determined using probit analysis.

### 2.7. Teratogenicity Assay Using Zebrafish (*D. rerio)* Embryo

Teratogenicity assay dealt with examination of the physiological development abnormalities occurring in organisms throughout their lifespan. Positioned within medical genetics as a specialized field, it concentrates on categorizing congenital abnormalities in dysmorphology attributed to teratogens. These teratogens are substances capable of inducing nonheritable birth defects through their toxic impact on an embryo or fetus [19]. The teratogenicity assay was adapted from Lindain et al. [18] with some modifications.

In a glass aquarium, an adult female and male zebrafish at a ratio of 1 female to 2 males were acclimatized for one week at 26 ± 1°C (room temperature). Mature zebrafish were placed in a coarse plastic mesh submerged within the aquarium water. This measure aimed to shield the eggs from potential predation by the mature zebrafish after fertilization. To induce spawning, the aquarium was covered with a black bag to maintain darkness for 12 h. The eggs were collected, and the morphological uniformity assessment was done before teratogenicity assay; unfertilized coagulated and ruptured eggs were discarded. The ethanolic extracts were diluted using embryo water (Hank’s solution). In each well of ELISA plates, 10 embryos were introduced alongside 3 mL of extract at varying concentrations. Mortality and morphological abnormalities were determined using a compound microscope at 12, 24, 36, and 48 h following the application of treatment.

### 2.8. Statistical Analysis

The experiment was laid out using a completely randomized design (CRD). The test for difference was done using analysis of variance (ANOVA) and comparison among means by post hoc test of homogenous subsets through Tukey’s HSD test. For cytotoxicity, the medium lethal concentrations (LC_50_ values) of the different ethanolic extracts were computed using the probit analysis. A significant level of difference was set at 0.05 level of significance.

## 3. Results and Discussion

### 3.1. Phytochemical Composition

All 28 plant samples were screened for the presence of their phytochemical constituents using TLC. Twelve phytochemicals were detected such as essential oils, triterpenes, phenols, fatty acids, sugar, anthroquinones, coumarins, anthrone, tannins, flavonoids, steroids, and alkaloids. As presented in Table 1, all the phytochemicals, except sugar and steroids were detected in all plant samples. Sugar was not detected in *C. grandis, M. pendula, R. humilis, A. brasiliana, M. calabura, P. foetida, R. simplex,* and *R. tuberosa*, while steroids were not detected in four species of *Curcuma* (*C. zedoaria, C. heyneana, C. mangga*, and *C. zanthorriza*) and in *Centella asiatica.* Using Borntrager’s reagents, the presence of anthraquinones was indicated by the formation of red zones; yellow zones for anthrones and blue fluorescence under UV (365 nm) for the presence of coumarins. Meanwhile, potassium ferricyanide–ferric chloride reagents resulted to blue–black zones for phenolic compounds and dark green for tannins. For alkaloids, using Dragendorff’s reagent, the formation of orange to red precipitate indicates its presence, and for the flavonoids, yellow to orange fluorescence under UV (360 nm) using Antimony III chloride. Lastly, for the detection of sugar, diphenylamine–aniline–phosphoric acid was used, wherein positive reactions produce the blue to violet spots [20–22].

**Table 1:** Phytochemical constituents of the selected medicinal plants.

Plant samples	Essential oils	Triterpenes	Phenols	Fatty acids	Sugar	Anthraquinones	Coumarins	Anthrones	Tannins	Flavonoids	Steroids	Alkaloids
*M. paniculata*	+	+	+	+	+	+	+	+	+	+	+	+
*K. angustifolia*	+	+	+	+	+	+	+	+	+	+	+	+
*Z. americana*	+	+	+	+	+	+	+	+	+	+	+	+
*P. cablin*	+	+	+	+	+	+	+	+	+	+	+	+
*C. asiatica*	+	+	+	+	+	+	+	+	+	+	+	+
*N. arbor-tris tis*	+	+	+	+	+	+	+	+	+	+	+	+
*S. arvensis*	+	+	+	+	+	+	+	+	+	+	+	+
*C. heyneana*	+	+	+	+	+	+	+	+	+	+	+	+
*B. pandurata*	+	+	+	+	+	+	+	+	+	+	+	+
*C. mangga*	+	+	+	+	+	+	+	+	+	+	—	+
*C. zedoaria*	+	+	+	+	+	+	+	+	+	+	+	+
*V. officinalis*	+	+	+	+	+	+	+	+	+	+	—	+
*T. paniculata*	+	+	+	+	+	+	+	+	+	+	+	+
*S. rhombifolia*	+	+	+	+	+	+	+	+	+	+	+	+
*P. crocatum*	+	+	+	+	+	+	+	+	+	+	+	+
*A. mangium*	+	+	+	+	+	+	+	+	+	+	+	+
*Z. zerumbet*	+	+	+	+	+	+	+	+	+	+	+	+
*C. zanthorriza*	+	+	+	+	+	+	+	+	+	+	—	+
*C. grandis*	+	+	+	+	+	+	+	+	+	+	+	+
*P. foetida*	+	+	+	+	+	+	+	+	+	+	+	+
*M. pendula*	+	+	+	+	+	+	+	+	+	+	+	+
*R. huminis*	+	+	+	+	—	+	+	+	+	+	+	+
*A. brasiliana*	+	+	+	+	—	+	+	+	+	+	+	+
*M. rubus*	+	+	+	+	+	+	+	+	+	+	+	+
*R. tuberosa*	+	+	+	+	—	+	+	+	+	+	+	+
*R. simplex*	+	+	+	+	+	+	+	+	+	+	+	+
*M. calabura*	+	+	+	+	—	+	+	+	+	+	+	+
*L. camara*	+	+	+	+	+	+	+	+	+	+	+	+

This coincides with previous studies revealing the presence of various secondary metabolites that support their medicinal importance [22, 23]. Plants belonging to *Zingiberaceae* contains bioactive compounds including curcuminoids such as volatile oils, flavonoids, phenolic compounds, curcumin, demethoxycurcumin, and xanthorrhizol [24, 25]. According to Kustina et al. [26] and Sánchez et al. [27], *V. officinalis* may contain sesquiterpenes (valerenic acid, valerenal, and related compounds) and valepotriates. *S. rhombifolia* leaf extracts are rich in flavonoids, tannins, and other polyphenols, isoquercitrin, apigenin, and kaempferol [28]. Additionally, *P. cablin* contains patchouli alcohol, α‐patchoulene, β‐patchoulene, α‐bulnesene, seychellene, norpatchoulenol, pogostone, eugenol, and pogostol [29]; *C. asiatica* contains asiaticoside, madecassoside, asiatic acid, and madecassic acid [30]; *P. foetida* has flavonoids such as vitexin, isovitexin, and orientin [31], while the members of family *Cucurbitaceae* have cucurbitacins which is a bitter tetracyclic triterpenoids [32].

### 3.2. Total Phenolics and Antioxidant Activity

**Figure 1:**
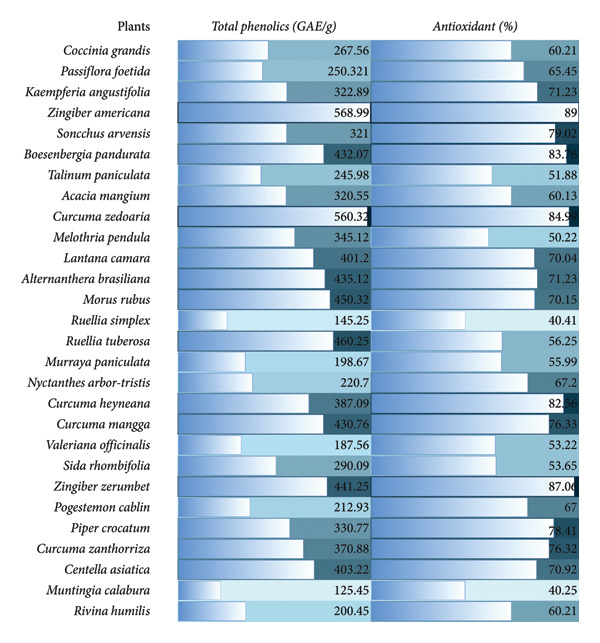
Heatmap of the total phenolics and antioxidant activity of the different plant extracts.

The total phenolic content of the selected plants ranges from 125.45 to 568.99 GAE/g, which were recorded from *M. calabura* and *Z. americana*, respectively (Figure 1). It can also be depicted that plant species such as *C. zedoaria* (560.32 GAE/g), *B. pandurata* (432.07 GAE/g), *A. brasiliana* (435.12 GAE/g), *M. rubus* (450.32 GAE/g), *R. tuberosa* (460.25 GAE/g), *C. mangga* (430.76 GAE/g), *Z. zerumbet* (441.25 GAE/g), and *C. asiatica* (403.22 GAE/g) had high total phenolic content. The detected phytochemicals and total phenolic content can contribute to the antioxidant activity of the selected plants. Accordingly, total phenolic contents of < 50 m gGAE/g is considered low with weak antioxidant activity; 50–100 as moderate with antioxidant potential and 100–250 as high with strong antioxidant activity [33–35].

The highest antioxidant activity of 87.06% was recorded in *Z. zerumbet*, followed by *C. zedoaria* (84.99%), *B. pandurata* (83.76%), and *C. heyneana* of 82.56%. The least antioxidant activity was recorded from *M. calabura* with 40.25%. Extracts with > 80% inhibition at a defined test concentration is generally considered to have strong antioxidant potential, 50%–80% moderate antioxidant effect and extracts with < 50% inhibition are considered to have lower antioxidant activity. Depending on the extraction method and assay used, the antioxidant capacity is reported as moderate to high, supporting its traditional use as a natural source of free radical scavengers [33–36].

This antioxidant potential is likely due to the combined action of its phenolic and flavonoid constituent of the plants [36, 37]. In addition, its polyphenolic constituents and essential oil components may help attenuate oxidative stress that may provide significant protection against free radicals and have potential benefits in reducing risks of chronic diseases such as cardiovascular disease, cancer, and neurodegeneration [38, 39]. And this supports the traditional use of the plant extracts against oxidative stresses [40].

### 3.3. Brine Shrimp *(A. salina)* Cytotoxicity

**Figure 2:**
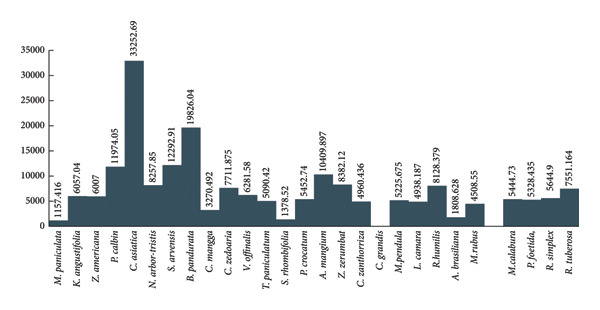
Lethality concentration at 50 of different extracts for brine shrimp cytotoxicity assay.

Brine shrimp cytotoxicity test was done using 5000, 1000, 500, and 100 ppm of plant extracts, and the lethality concentration (LC_50_) at 24 h of incubation was determined. As shown in Figure 2, the cytotoxicity of each plant extracts are dose‐ and time‐dependent. Whereas increasing the concentration of the plant extracts and the time of exposure also increased the mortality rate of the brine shrimp. The same is true with the finding of Sharma et al. [41], wherein many plant extracts exhibit dose‐dependent cytotoxicity. According to Saleem et al. [42], Cao et al. [43], and Panche et al. [44], cytotoxicity can be due to the presence of the phytochemicals that induce apoptosis, cell membrane disruption, and generation of reactive oxygen species. This includes phytochemicals such as essential oil, flavonoids, and other phenolic compounds [45]. Also, certain polyunsaturated fatty acids (PUFAs) have been shown to exert cytotoxic effects, can induce lipid peroxidation, alter membrane fluidity, and trigger apoptotic pathways [19]. Many coumarins also show moderate cytotoxic activity in tumor cells [10]. Lastly, alkaloids represent some of the most potent cytotoxic compounds by interfering with microtubule dynamics, DNA intercalation, and topoisomerase inhibition, leading to cell cycle arrest and apoptosis [3].

**Figure 3:**
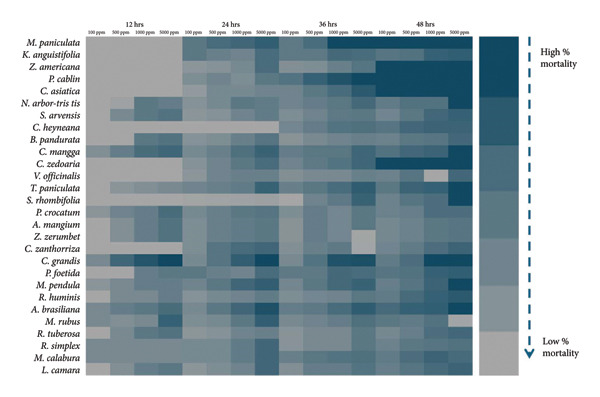
Cytotoxicity of the plant extracts at 12, 24, 36, and 48 h of incubation in brine shrimp (*Artemia salina*).

Figures 2 and 3 present the LC_50_ value of the plant extracts and the mortality of brine shrimp exposed to different concentrations of plant extracts. According to Clarkson et al. [46], LC_50_ of 0–100 ppm are highly toxic, 100–500 ppm as medium toxic, 500–1000 ppm as low toxic, and above 1000 μg/mL as nontoxic. Based on the results, only *C. grandis* is toxic, with an LC_50_ value of 807 ppm. From the computed LC_50_, only *C. grandis* with LC_50_ of 807.70 ppm at 24 h of incubation and the rest of the plant extracts can be considered as nontoxic at 24 h of incubation. The results of the study also coincide with the previous studies that some of the selected plants are noncytotoxic such as *M. pendula* [47], *P. foetida* [48], *Z. americana, C. mangga* [25], *C. zanthorriza* [49], *V. officinalis* [50], *S. rhombifolia* [51, 52], *P. cablin* [52], *M. calabura* [53], *R. humilis* [54], *C. asiatica* [55], *M. rubus* [56], *M. paniculata* [57, 58], *S. arvensis* [59], *T. paniculata* [60], *A. mangium* [61], *L. camara* [62], and *Nyctanthes arbor-tristis* [63].

The noncytotoxicity of the screened plants is generally favorable for medicinal use especially for therapies intended for long‐term use or systemic administration. Noncytotoxic extracts are less likely to harm healthy cells, making them safer for therapeutic use. A wide margin between effective dose and toxic dose improves clinical viability. It is considered safer for long‐term administration in conditions such as inflammation, diabetes, or neurodegeneration. Noncytotoxic compounds may still exhibit strong antioxidant, anti‐inflammatory, or antimicrobial effects [60, 61, 63]. They can be further fractionated and tested in more advanced assays, ultimately supporting their potential development as pharmaceutical or nutraceutical agents with favorable safety profile.

### 3.4. Morphological Abnormalities and Teratogenicity Effects of Plant Extracts in Zebrafish (*D. rerio)*

**Figure 4:**
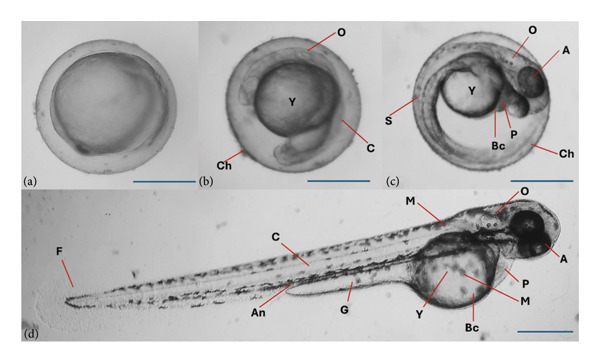
Normal zebrafish embryogenesis. (a) Blastula period; (b) segmentation period (24 h); (c) Pharyngula period (48 h); (d) hatching period. A: enlarged eye; An: anus; Bc: blood cells; C: chorda; Ch: chorion; F: fin; G: gut; M: melanophores; O: ear bud; P: pericard; S: somites; Y: yolk sac.

Teratogenic effects were assessed through parameters such as growth retardation, restricted movement, malformations, pigmentation, and coagulation. In Figure 4, the different stages of a normal zebrafish embryogenesis from blastula stage to hatching stage are shown. During the blastula stage, the blastocyst may contain about 256 blastoms. For 2–5 h, gastrula period also occur wherein epibolic movement takes place where blastoderm become curved. At segmentation period, the somites development, muscular twitches, and end tail extension can also be observed. In the pharyngula stage, spontaneous movement with detachment of the tail from the yolk is visible. Pigmentation can reduce the movement. During the hatching period, which can occur between 48 and 76 h.

**Figure 5:**
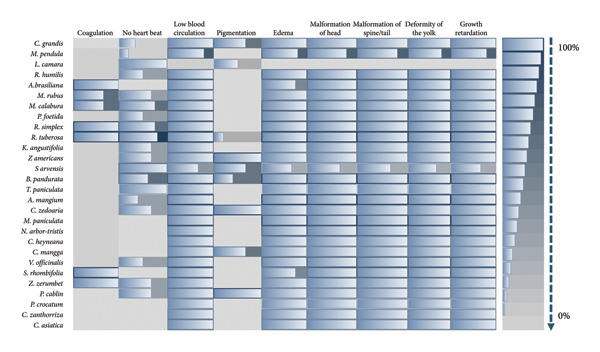
Heatmap for the presence of different toxicological and teratogenic defects in zebrafish embryo.

Various morphological abnormalities and teratogenic effects were observed in different developmental stages of zebrafish when exposed to 5000, 1000, 500, and 100 ppm of plant extracts. Lethal and sublethal endpoints for evaluating the toxicity and teratogenicity as noted by Nagel were used. Toxicological endpoints, as presented in Figure 5, can be lethal and sublethal. Lethal effects predominantly by coagulation of the embryo was observed during the 12 h of exposure to different plant extracts. While at 24 h, reduced or lack of heartbeat and blood circulation and delayed growth are noticeable. Pericardial and yolk sac edema are also visible. Meanwhile for the teratogenic effect, malformations of the head, notochord, and tail were observed. Slow to no heartbeat, growth retardation, and mummification were also noted. Among the plant used, *L. camara* produced the least teratogenic effects of no heartbeat and pigmentation.

Coagulation of the zebrafish embryo was observed in *A. brasiliana, M. rubus, M. calabura, R. simplex, R. tuberosa, S. rhombifolia,* and *Z. zerumbet*. An opaque and coagulated embryo is considered as an early sign of fatal development failure, while failure to development somites means disruption in the early segmentation and somatogenesis can lead to nonviability. Additionally, nondetachment of the tail can be due to development arrests and could result in mortality [64–66]. For the sublethal effects, pericardial edema indicates cardiac dysfunction and can lead to abnormal accumulation of fluid around the heart [65, 66]. Yolk sac edema or the swelling of the yolk sac area is associated with the disrupted osmoregulation and general developmental stress, while tail malformation may indicate musculoskeletal or neural developmental disruptions [64]. Also, the delayed hatching can be due to related toxic substances, and abnormal pigmentation, such as changes in pigment distribution, is a sensitive marker of sublethal developmental toxicity [65].

Malformations in zebrafish embryos such as head, tail, and spine deformities when exposed to plant extracts are strong indicators of teratogenicity, cytotoxicity, and developmental toxicity. These effects often stem from bioactive secondary metabolites such as alkaloids, flavonoids, tannins, and terpenoids. Head malformation in zebrafish implies disruption of brain morphogenesis, craniofacial development, and neural crest migration. In which alkaloids can interfere with the neural tube closure [67] and phenolic compounds may inhibit signaling necessary for head development [68]. On the other hand, the presence of tail malformation may lead to abnormal posterior axis elongation, somite elongation, somite segmentation, and muscle patterning. This can be induced by oxidative stress and apoptosis in tailbud cells by the flavonoids such as quercetin and hesperidin [69]. Also, the saponins can cause cell adhesion and migration [66]. While the spine malformation which indicates defects in the notochord integrity, vertebral ossification and collagen synthesis can be attributed as toxic effects of terpenoids and tannins. Accordingly, terpenoids such as xanthones are linked to scoliosis and bent spine phenotypes [70], and tannins can cause the downregulation of genes affecting skeletal formation [71].

**Figure 6:**
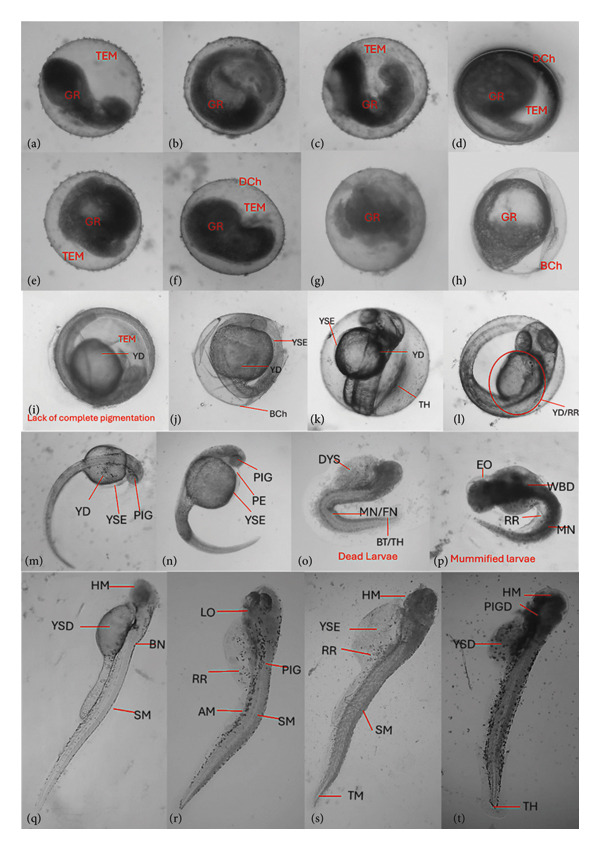
Malformation and teratogenic effects at 12, 24, and 48 hpf. GR: growth retardation; TEM: turbid embryo membrane; DCh: deformed chorion; BCh: broken chorion/ruptured membrane; YD: yolk sac deformation; YSE: yolk sac edema; YSD: yolk sac deformation; TH: tail hypoplasia; RR: reduced resorption; PIG: pigmentation; PE: pericardial edema; MN: notochord malformation; FN: fragmented notochord; BT: bent tail; EO: edema; HM: head malformation; SM: spine malformation; TH: tail malformation; DYS: disintegration of yolk sac; AM: axial malformation.

When zebrafish embryos exhibit a slow heartbeat, abnormal pigmentation, or coagulation after exposure to plant extracts, these are strong indicators of cardiotoxicity, melanogenic disruption, and embryo lethality. Slow heartbeat also known as bradycardia suggests cardio cytotoxicity, impaired calcium signaling, and mitochondrial dysfunction which can also be associated with reduced circulation, developmental delay, and increased mortality. These can be affected by the presence of alkaloids and phenolics. Alkaloids inhibit mitochondrial Complex I, reducing ATP and slowing cardiac rhythm and phenolics may interfere with ion channels, affecting cardiac contractility [72, 73]. Meanwhile, pericardial edema in zebrafish embryos implies fluid accumulation around the heart causing developmental disruption. Several secondary metabolites from plants can trigger pericardial edema due to their effects on cardiac morphogenesis, osmoregulation, and mitochondrial function [74]. Alkaloids impair ATP production, while phenolics disrupt ion channels and calcium signaling cardiac looping defects and edema [75, 76]. Terpenoids alter epidermal integrity and osmoregulation, while anthraquinones cause inhibition of tyrosinase and affect pigment and cardiac development [76, 77]. Changes in pigmentation can be attributed to the disruption of melanogenesis, tyrosinase activity, or pigment cell differentiation as affected by flavonoids, terpenoids, and anthraquinones. Flavonoids and anthraquinones inhibit tyrosinase, leading to hypopigmentation, and terpenoids may downregulate melanin synthesis genes [75–77]. Lastly, coagulation of embryos is a hallmark of embryo lethality, often due to cytotoxicity or severe teratogenicity by saponins, alkaloids, and tannins (Figure 6). Saponins and alkaloids can disrupt membrane integrity, and tannins induce oxidative stress and apoptosis in early embryonic stages [78].

**Figure 7:**
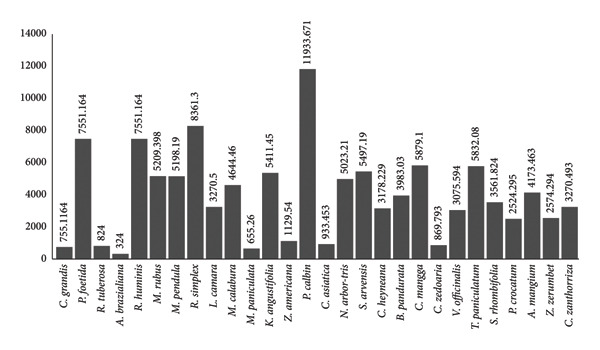
LC_50_ in ppm for the teratogenicity of the plant extracts.

In zebrafish teratogenicity, a compound is generally considered highly toxic if it shows LC_50_ values below about 100 ppm, indicating that even low concentrations can cause significant mortality coupled with developmental defects (pericardial edema, spinal curvature, and craniofacial defects). Conversely, compounds with LC_50_ values greater than 1000 ppm tend to be regarded as having low acute developmental toxicity [79]. As shown in Figure 7, only *P. foetida, A. brasiliana*, and *P. cablin* had LC_50_ values < 1000 ppm but not > 100 ppm, and the rest had the LC_50_ > 1000 ppm. Thus, this study revealed that all 28 tested plant extracts can still be considered as nonteratogenic causing mild developmental toxicity to the zebrafish.

Reports are still lacking on the teratogenicity of some of the tested plants since majority of which are used in culinary and in medicine. According to Sutrisno and Kröner [80], Chahyadi et al. [81], and Hidayat et al. [82], most of the plant species belonging to Zingiberaceae are generally considered safe; however, very high doses could cause development delays. In previous studies, exposure of zebrafish and rodents to *B. pandurata* and *Z. zerumbet* can cause developmental disruptions and skeletal malformations [81, 83, 84]. Similarly, *V. officinalis* at high dose can cause skeletal change [84]. However, generally at low concentrations, all plant extracts are considered safe.

## 4. Concluding Remarks

Based on the findings, all 28 species of selected plants contain phytochemicals, with considerably high total phenolic content which contributes to their high antioxidant activity. Based on the results, the selected plants were considered noncytotoxic and with low‐ to nonteratogenic potential. Thus, further research must be done to determine which of the promising plants have good activity and no toxic effects.

## Conflicts of Interest

The authors declare no conflicts of interest.

## Author Contributions

All the authors contributed equally to conceptualization, experimentation, analysis, and writing of this research.

## Funding

The authors would like to thank the Airlangga Post Doctoral Fellowship Program, Universitas Airlangga, for funding this research project with contract number 405/B/UN3.AGE/HK.07.01/2025 and International Research Network with the contract number 3115/B/UN3.LPPM/PT.01.09/2024.

## Data Availability

The data used to support the findings of this study are included within the article.
